# The Signature of Moderate Perinatal Hypoxia on Cortical Organization and Behavior: Altered PNN-Parvalbumin Interneuron Connectivity of the Cingulate Circuitries

**DOI:** 10.3389/fcell.2022.810980

**Published:** 2022-02-28

**Authors:** Sara Trnski, Barbara Nikolić, Katarina Ilic, Matea Drlje, Mihaela Bobic-Rasonja, Sanja Darmopil, Zdravko Petanjek, Dubravka Hranilovic, Natasa Jovanov-Milosevic

**Affiliations:** ^1^ Croatian Institute for Brain Research, School of Medicine, University of Zagreb, Zagreb, Croatia; ^2^ Department of Biology, Faculty of Science, University of Zagreb, Zagreb, Croatia; ^3^ Department of Neuroimaging, BRAIN Centre, Institute of Psychiatry, Psychology, and Neuroscience, King’s College London, London, United Kingdom; ^4^ Department of Biology, School of Medicine, University of Zagreb, Zagreb, Croatia; ^5^ Department of Anatomy and Clinical Anatomy, School of Medicine, University of Zagreb, Zagreb, Croatia

**Keywords:** plasticity, oxidative stress, cortical development, learning disabilities, hyperactivity behaviors

## Abstract

This study was designed in a rat model to determine the hallmarks of possible permanent behavioral and structural brain alterations after a single moderate hypoxic insult. Eighty-two Wistar Han (RccHan: WIST) rats were randomly subjected to hypoxia (pO2 73 mmHg/2 h) or normoxia at the first postnatal day. The substantially increased blood lactate, a significantly decreased cytochrome-C-oxygenase expression in the brain, and depleted subventricular zone suggested a high vulnerability of subset of cell populations to oxidative stress and consequent tissue response even after a single, moderate, hypoxic event. The results of behavioral tests (open-field, hole-board, social-choice, and T-maze) applied at the 30–45th and 70–85th postnatal days revealed significant hyperactivity and a slower pace of learning in rats subjected to perinatal hypoxia. At 3.5 months after hypoxic insult, the histochemical examination demonstrated a significantly increased number of specific extracellular matrix—perineuronal nets and increased parvalbumin expression in a subpopulation of interneurons in the medial and retrosplenial cingulate cortex of these animals. Conclusively, moderate perinatal hypoxia in rats causes a long-lasting reorganization of the connectivity in the cingulate cortex and consequent alterations of related behavioral and cognitive abilities. This non-invasive hypoxia model in the rat successfully and complementarily models the moderate perinatal hypoxic injury in fetuses and prematurely born human babies and may enhance future research into new diagnostic and therapeutic strategies for perinatal medicine.

## Introduction

Perinatal cerebral hypoxia-ischemia and preterm birth are the most common causes of neurological disorders or impaired brain function of developmental origin (Volpe, 2012; Vohr, 2014; Laptook, 2016; Vanes et al., 2021). Although current advances in medicine provide good perinatal care, 30% of premature babies who survive still suffer from neurodevelopmental motor, cognitive, or behavioral deficits in childhood or later in life (Johnson, 2011; Franz et al., 2018). Despite the multifactorial and combinatorial etiology (genetic, trophic, infectious, inflammatory, ante- or postnatally), the most frequently found periventricular white matter injury, commonly results from hypoxic-ischemic reperfusion failure (Liu et al., 2013; Clowry et al., 2014; Millar et al., 2017). The highest vulnerability of the human brain to hypoxic injuries is between 22- and 34 weeks of gestation, at the stage of intensive cell migration, glial cell proliferation, axon guidance, synaptogenesis, and dendrite and connectivity elements differentiation. In addition to periventricular regions, the axonal crossroads and subplate zone with an abundance of extracellular matrix and transient circuitries are at particular risk (Kostović et al., 2014a; Kostović et al., 2014b; Milošević et al., 2014; Milos et al., 2020).

A “gold standard” of animal models in performing hypoxic-ischemic brain injury is the Rice-Vannucci model and its numerous modifications (Rice et al., 1981; Vannucci et al., 1999; Millar et al., 2017). In rodents, the time frame from postnatal day (P) 1 to P3 is approximately equivalent to the period of 22–32 weeks of gestation (wg) for human brain development, based on morphogenetic processes, such as proliferation and migration, while P7-10 approximately correspond to 32–40 wg according to vasculogenesis and time of six-layered cortex formation. P40 approximately corresponds to the maturation of interneurons and approximately P60, corresponds to early adulthood cortex (Semple et al., 2013).

Depending on the developmental stage of the animal and the intensity of the provoked hypoxia, the extent of the neuronal dysfunction in these models ranges from severe, with significant pathology that intercepts most of the developmental processes suddenly (including ischemia infarction, hemorrhage necrosis, inflammation with cytokine secretion, massive activation of microglia and astroglia, scarification), to less severe lesions such as disorganized barrels field in the somatosensory cortex, focal neuronal loss, and edema (Rice et al., 1981; Vannucci et al., 1999; Quairiaux et al., 2010; Zhang et al., 2013). Due to the severity of the injury, an acutely increased expression of cleaved caspase-3 protein and hypoxia-inducible factor-1α (Hif-1α) in addition to deficits in spatial reference memory, disturbed motor, and auditory abilities, have been reported (Huang et al., 2013; Alexander et al., 2014; Takada et al., 2015; Sukhanova et al., 2018).

However, although many studies have used these animal models, studies of moderate hypoxic injury and its long-term effects, especially at the brain structural (histological) and functional (behavioral) level, are missing or scant (Clowry et al., 2014; Millar et al., 2017). The moderate hypoxic events in human fetuses during the midgestation period, frequently remain unnoticed. However, they might induce consequent processes leading to medical issues in vulnerable and predisposed individuals, ending with pregnancy termination or the birth of extremely premature born babies. In less susceptive subjects, they might lead to long-term brain structural or behavioral alterations. Most of the postmortem human brain tissue received from pathology departments and used in translational studies is staged 18–30 weeks of gestation. Thus, for translational studies in perinatal medicine, developing a corresponding P1 model, complementary to other animal later period models, could be critical.

Some moderate hypoxia events in fetuses and prematurely born babies (unnoticed acutely) are recognized later as a cause of cognitive or behavioral deficits during early school days or adolescence. These deficits are related to a developmental hypoxic incident that causes moderate to subtle white matter or cortical microstructure pathology (Johnson and Marlow, 2011; Kelly et al., 2016; Batalle et al., 2017; Ball et al., 2020).

The interneurons and gamma-aminobutyric acid (GABA) signaling are recognized as a common substrate of the perinatal injuries that underlie many neurodevelopmental disorders (Deidda et al., 2014). The interneurons make up 10–30% of cortical neurons (Defelipe et al., 2013), more represented in humans than in rodents. Approximately 40% of all GABA-ergic neurons express parvalbumin protein, which are the main interneuron population in rodents (Rudy et al., 2011). Parvalbumin neurons are mainly fast-spiking GABA-ergic neurons that have a fundamental role in the moderation of excitation and inhibition within cortical circuits (Ferguson and Gao 2018).

The parvalbumine-postive interneurons (PV) are found to be affected in many neurological and psychiatric disorders of developmental origin in humans and in the animals that model these disorders (Gandal et al., 2012; Filice et al., 2016; Hashemi et al., 2018; Vogt et al., 2018). The PV frequently have a specialized, condensed, extracellular matrix coating called perineuronal nets (PNN), which is known to have a modulating role in GABA signaling and neural plasticity after injury (Bitanihirwe and Woo, 2014; Fawcett et al., 2019). The experience-dependent synaptic plasticity in adulthood also relies particularly on PNN around fast-spiking PV (Wang and Fawcett, 2012; Sorg et al., 2016). Several preclinical studies have analyzed PNN after acute hypoxic brain injury (Härtig et al., 2017; Stolp et al., 2019). However, the long-term effects of perinatal hypoxia incidents, particularly the mild to moderate effects, on PNN—interneurons development and consequent brain function, have not been investigated. However, disclosing molecular and cellular cause-consequence sequela, the time points of highest vulnerability, and their relation to neurological and cognitive outcome, could significantly improve our understanding of perinatal hypoxia and enhance the search for potential therapeutic targets.

We hypothesized that the development of the PNN—interneuron complex is highly vulnerable, even to a single, moderate hypoxic event. We aim to investigate the effect of moderate hypoxia on early neonatal development in a rat model (P1, which corresponds to the mid-fetal and early premature period in humans), in the acute phase at the molecular and cellular levels and in the chronic time frame, examining the subsequent structural reorganization level. Finally, we correlated these changes with the functional consequences, investigating the behavioral outcome in juvenile and adult animals.

## Results

### Acute Molecular, Cellular, and Structural Brain Changes After Moderate Perinatal Hypoxia

**FIGURE 1:**
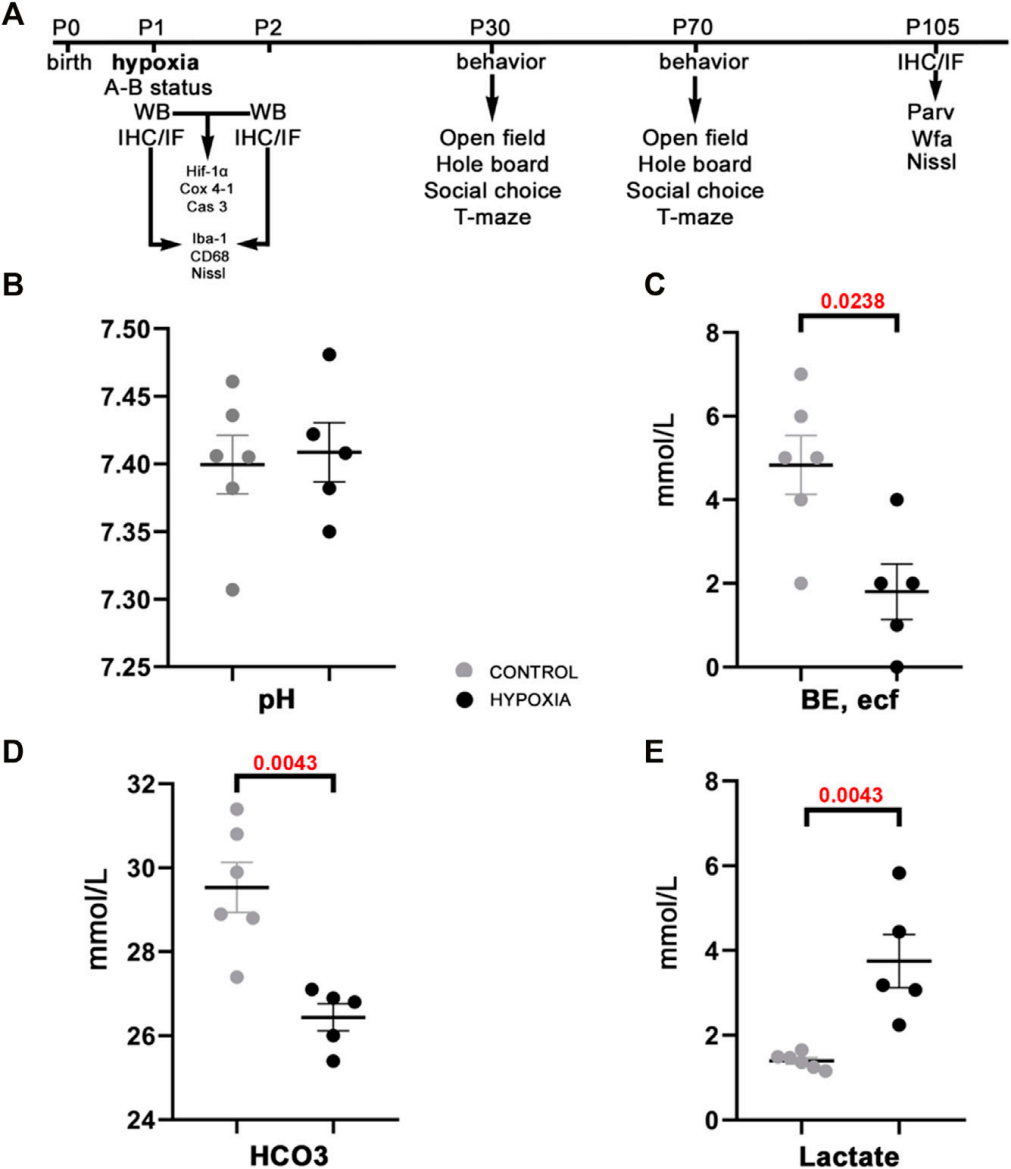
The study design and acid-base status of the animals indicate sufficient compensatory buffers capacity, ensuring acid-base homeostasis at this design of moderate perinatal hypoxia in rat. **(A)** A presentation of the study design by timeline with the time points chosen for data acquisition by various tests, staining’s of brain tissue sections, and subsequent qualitative and quantitative analysis of the behavior and brain structures. Treated and control animal distribution per each experiment is presented in Supplementary Table S1. The treated rats were subjected to hypoxia (during 2 h) at P1. Immediately after, some were sacrificed to measure different parameters of A-B status. Other animals were sacrificed later at 8 h, and 24 h after hypoxia for brain samples, on which WB, IF, IHC were employed with different antibodies. The antibodies used in the study are listed in Table 1. The remainder of the animals were tested at two different age points: starting at P30 and P70 during five subsequent days. The animals were tested with four behavioral tests (open filed, hole board, social choice, T-maze). These animals were sacrificed at P105 to isolate the brain tissue for differential staining’s and further data acquisition. **(B–E)** The values of different blood parameters showing acid-base status measured immediately after hypoxia. **(B)** The pH (hydrogen potential) values speak in favor of sufficient compensatory capacity that ameliorates electrolyte imbalance in the rat neonates. **(C)** BE, ecf (base excess in the extracellular fluid) show significantly lower values in the hypoxic animals due to depletion of base buffers for compensation of the metabolic acidosis. **(D,E)** The HCO_3_^−^ (bicarbonate) and lactate concentrations in the blood show statistically significant differences between hypoxia-treated and control animals, proving shift from aerobic to anaerobic metabolic condition in the tissue as a consequence of hypoxia. All results are shown as mean ± standard error of the mean (SEM). P-postnatal day; A-B—acid-base status; WB-Western blot; IHC/IF—immunohistochemistry and/or immunofluorescence; Hif-1α—hypoxia-inducible factor 1α; Cox 4-1—cytochrome c oxidase subunit 4 isoform 1; Cas 3—caspase 3; Iba-1—ionized-calcium-binding-adaptor-molecule-1; CD68—class D scavenger receptor 68; Nissl (modification of cresyl-violet) staining; Parv—parvalbumin; Wfa—Wisteria floribunda agglutinin.

Immediately after hypoxic conditions were terminated, we measured the acute metabolic response by assessing the acid-base status in treated and control animals. The assessment of the acid-base status has confirmed the metabolic shift, a statistically significantly lower BE, ecf (*p* = 0.0238; Figure 1C), lower total HCO_3_^−^ (*p* = 0.0043; Figure 1D), and higher lactate concentrations (*p* = 0.0043; Figure 1E) in the blood of hypoxic animals as a consequence of acute generalized hypoxic stress. Even though pups showed restlessness, shortness of breath, convulsive twitching of the whole body, and general cyanosis during the hypoxia (video available upon request), the pH blood values were maintained within the physiological range (Figure 1B), and shortly after the hypoxia, pups looked seemingly healthy and behaved age-appropriately. These findings suggest a sufficient extracellular buffers capacity and rapid achievement of acid-base homeostasis after the hypoxic incident in the pups.

**FIGURE 2:**
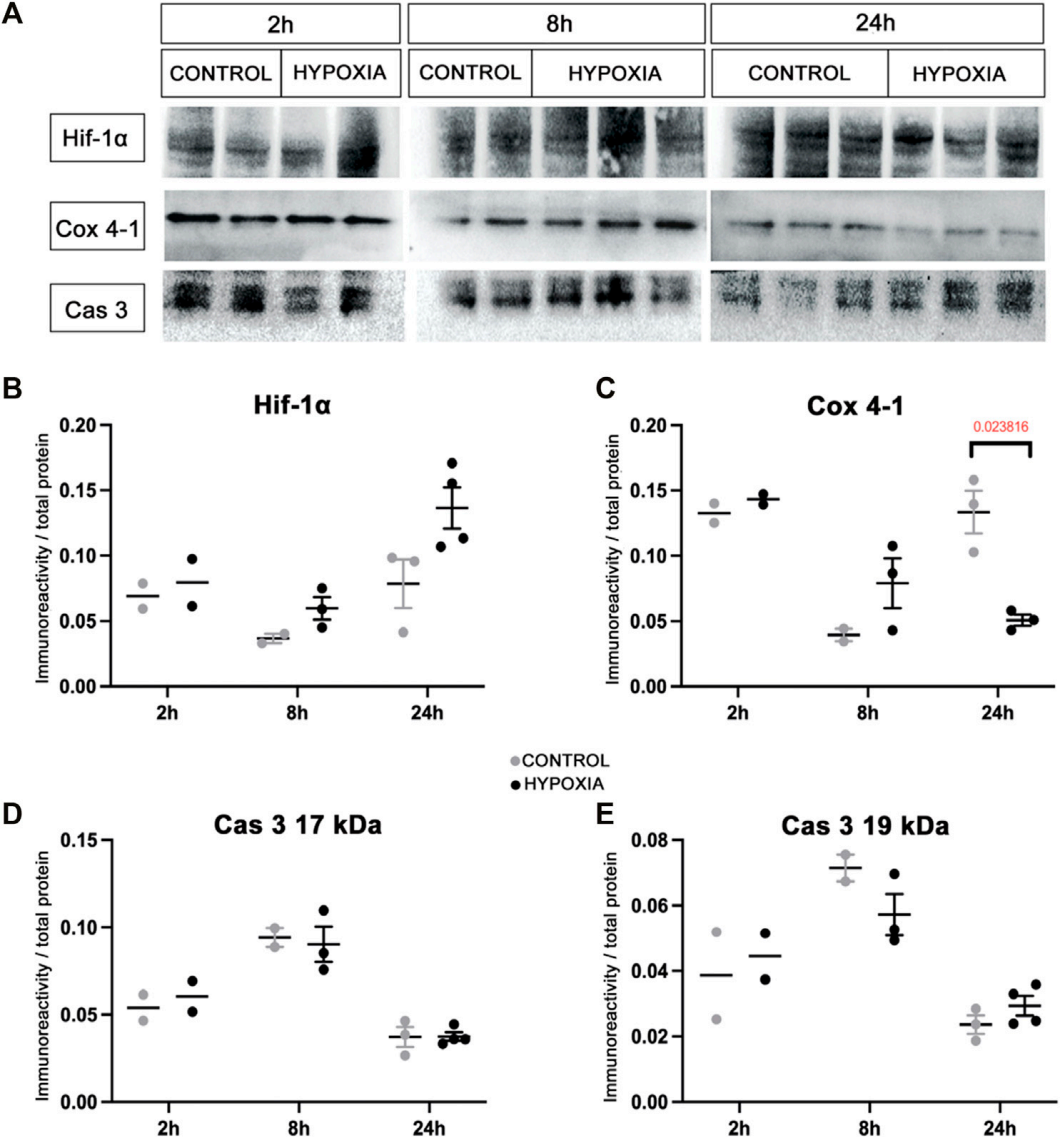
Expression of the hypoxia-related proteins in the brain tissue of control and hypoxia-treated animals confirms moderate hypoxic lesion. **(A)** Representative Western blots of hypoxia-inducible factor-1 alpha (Hif-1α), cytochrome C oxidase subunit-4 isoform-1 (Cox 4-1), and cleaved caspase 3 (Cas 3) from brain samples obtained at 2, 8, and 24 h post hypoxia. The results of quantification of protein signal intensity is shown in correspondence to total protein amount (in Suplementary material suppl. Figure 1). **(B)** Notable changes in expression of Hif-1α (control 0.078 ± 0.011, hypoxia 0.136 ± 0.008), and **(C)** Cox 4-1 (control 0.133 ± 0.009, hypoxia 0.051 ± 0.003, *p* = 0.024, Student’s *t-*test) were measured in the samples obtained 24 h after the hypoxia. There is no difference between the groups at earlier time points. **(D,E)** There is no observed difference in Cas 3 expression in the brain tissue between the control and hypoxia group at any time point.

At the brain tissue molecular level, alterations in the expression of hypoxia-inducible factor-1-alpha (Hif-1α) and the expression of the cytochrome-c-oxidase subunit-4 isoform-1 (Cox 4-1) were observed (Figure 2A). The increase in the expression of Hif-1α protein was most notable at 24 h post hypoxia (Figure 2B). In addition, a gradual decrease of the Cox 4-1 protein expression was observed, becoming statistically significant at 24 h after hypoxia (*p* = 0.0238; Figure 2C). No difference in the expression of the activated caspase-3 protein (Cas 3) was detected between the two groups (Figures 2D,E).

**FIGURE 3:**
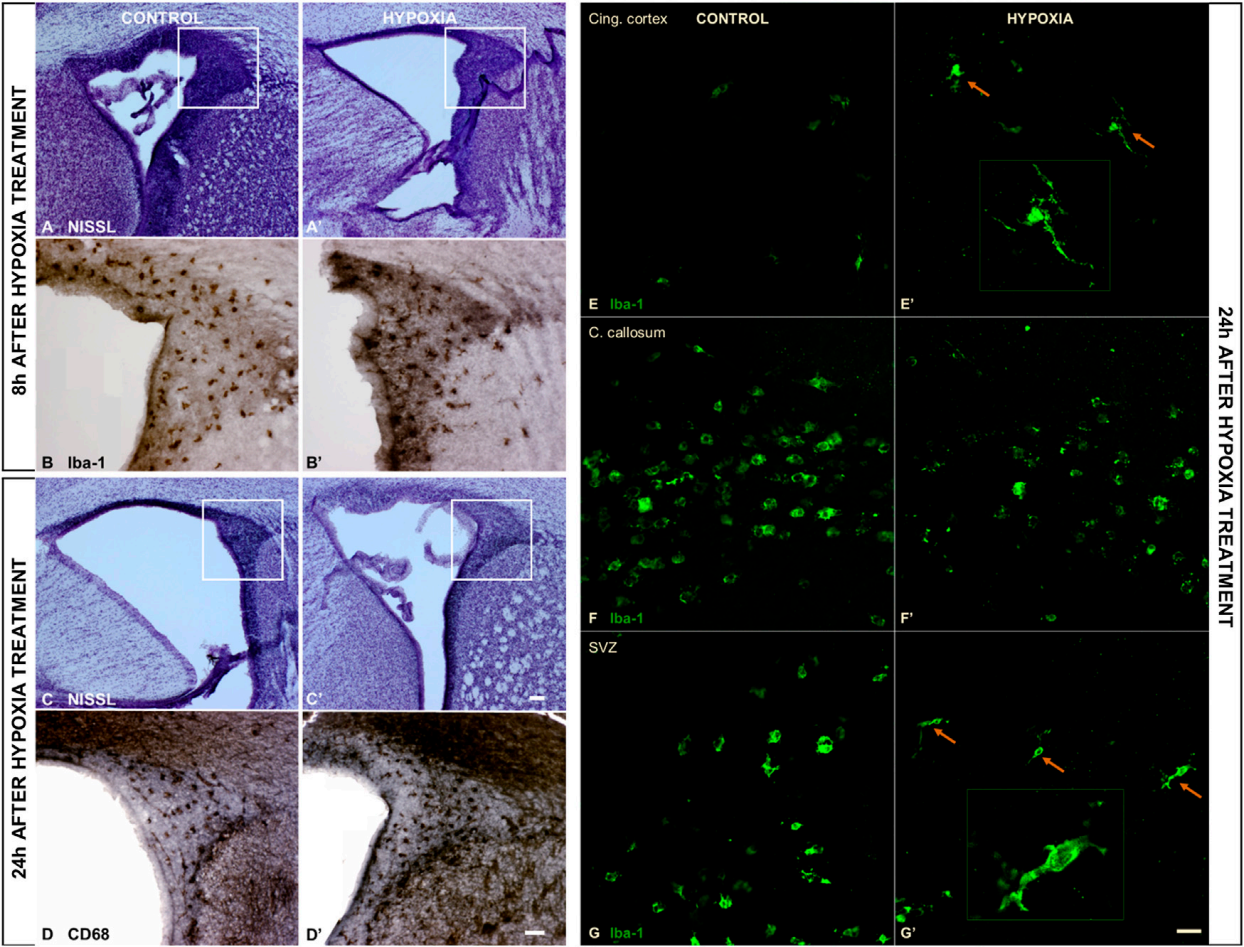
The coronal sections of the subventricular zone (SVZ) and microglial cell morphology confirming the affected proliferation pool and limited microglial cell activation after moderate perinatal hypoxia. **(A,A’,C,C’)** Show a distinct decrease of cell density in the proliferative SVZ in hypoxia-treated animals compared to controls, at 8 h **(A,A’)** and more pronounced at 24 h **(C,C’)** after hypoxia. **(B,B’)** The ionized-calcium-binding-adaptor-molecule-1 (Iba-1) staining 8 h after hypoxia shows less numerous but more ramified microglial cells in SVZ in hypoxia-treated animals. **(D,D’)** The class D scavenger receptor 68 (CD68) staining 24 h after hypoxia reveals no severe macrophage reactivity in hypoxia-treated animals compared to controls. **(E–G’)** Display a change in morphology of Iba-1-immunoreactive microglia in the cingulate cortex and SVZ, but not in the corpus callosum. Control animals’ cingulate cortex and SVZ contained intermediate microglia **(E,G),** while in hypoxia-treated animals, microglia developed a ramified morphology [**(E’,G’)** arrows], suggesting their precocious maturation. **(F,F’)** Within the corpus callosum, these cells have the same amoeboid morphology in both groups of animals. The actual image magnification for **(A,A’,C,C’)** is shown with the scale bar in **(C’)**, presenting 100 µm, for **(B,B’,D,D’)** is shown in **(D)** presenting 50 µm, and for **(E–G’)** scale bar is in **(G’)** presenting 25 µm.

An examination of the macromorphology of brains and sections stained by cresyl-violet (Nissl modification) showed an absence of gross pathological or anatomical changes. However, a lower cell density was observed in the subventricular zone (SVZ) 8 h post hypoxia (Figure 3A’), which was more pronounced 24 h post hypoxia (Figure 3C’) when compared with the SVZ of control animals (Figures 3A,C). The SVZ of hypoxia-treated animals also showed fewer ionized-calcium-binding-adaptor-molecule-1 immunohistochemically reactive cells (Iba-1), with increased content of Iba-1 protein in the immediate extracellular cell vicinity (Figure 3B’), compared to controls (Figure 3B). Despite an overall lower cell density in the SVZ at 24 h after hypoxia, we observed more class D scavenger receptor 68-expressing cells (CD68) in this zone, particularly its dorsal-lateral portion (Figure 3D’ compared to D). The Iba-1 positive cells showed an advanced differentiation at 24 h after subjection to hypoxia, acquiring the morphology of mature microglia in the cingulate cortex (Figures 3E,E’) and in the SVZ (Figures 3G,G’). There was no difference in the morphology of amoeboid Iba-1-positive cells in the corpus callosum between the two groups (Figures 3F,F’). Amoeboid microglial cells in both groups are probably engaged primarily in phagocytosis of the exuberant developmental axons in the corpus callosum. Altogether, examination of the histological sections revealed acute injury of proliferation processes in the SVZ and consequent mild microglial cell activation in this model of moderate perinatal hypoxia.

### Behavioral Alterations in Young and Adult Rats After Perinatal Hypoxia

**FIGURE 4:**
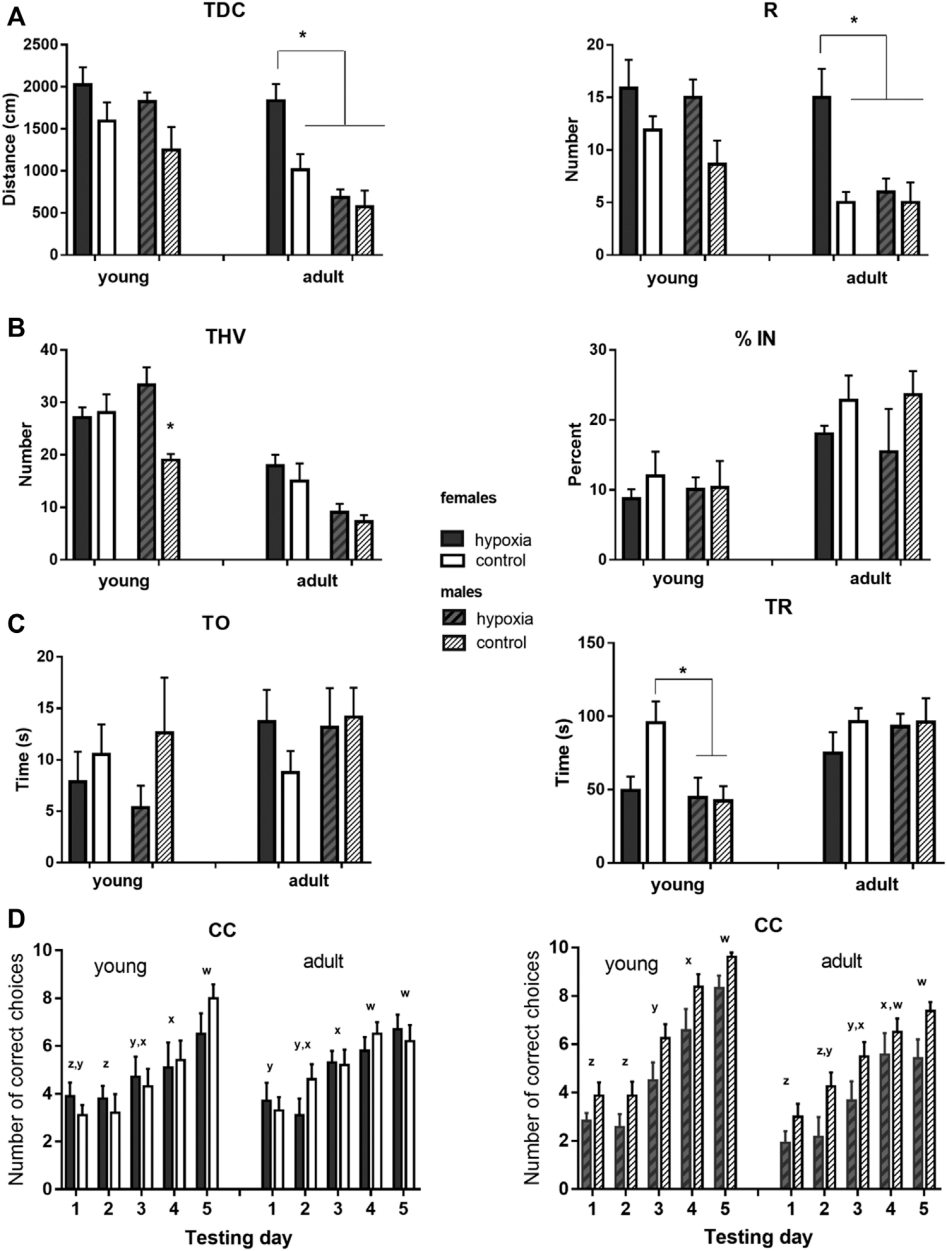
Results of the behavioral testing in young and mature animals display altered behavior and cognition in juvenile and adult rats. The rats were exposed to a battery of behavioral tests at juvenile (adolescent) (P33-P41) and adult (P83-P91) ages, to examine whether any behavioral and cognitive differences were still present or had newly appeared in adulthood. **(A)** Locomotor activity was measured as the total distance covered (TDC) and the number of rearing times (R) in an open field. Two-way ANOVA revealed: for TDC, a significant influence of hypoxia in juveniles and of hypoxia, sex, and hypoxia-sex interaction in the adults; for R, there was a significant influence of hypoxia in juveniles and a significant influence of hypoxia and sex, with the indicative influence of hypoxia-sex interaction in the adults. **(B)** Exploratory behavior was measured as the total number of visited holes (THV), and anxiety-like behavior was measured as the percentage of visited inner holes (% IN) in a hole-board. Two-way ANOVA revealed: for THV, a significant influence of hypoxia and hypoxia-sex interaction in juveniles and a significant impact of sex in adults; for % IN, no significant influences. **(C)** Social behavior was measured as time spent exploring an object (TO) and time spent exploring a rat (TR) in a social choice apparatus. Two-way ANOVA revealed for TO, no significant influences; for TR, a significant influence of sex, and hypoxia x sex interaction, only in juveniles. **(D)** Learning abilities were measured in a T-maze separately for males and females, and as the number of correct choices (CC) in sessions of ten trials during five consecutive days. Repeated measure two-way ANOVA revealed: for males, a significant influence of hypoxia and time, in juveniles, and an indicative influence of hypoxia and a significant effect of time, in adults; for females, the analysis showed a significant influence of time, in both juveniles and adults. Results are shown as mean ± standard error of the mean (SEM); Tukey’s honestly significance post-hoc test was performed after establishing a significant/indicative hypoxia-sex interaction influence and values that significantly differ are marked with an *(A–C). Letters x-z are indicators of significant differences (different letters) or the lack of significant difference (same letters) in the mean number of correct choices among different testing days as revealed by Tukey’s honestly significance post-hoc test **(D)**.

The results of behavioral testing are shown in Figure 4, with numerical values and statistical parameters given in the Supplementary material (Supplementary Tables 2, 3).

In juvenile animals, the open field test revealed a significant influence of hypoxia on both, horizontal (*p* < 0.01) and vertical (*p* < 0.05) activity, with hypoxic rats displaying higher mean values for TDC and R than control rats (Figure 4A). In adult animals, compared to the juveniles, mean values of the measured parameters decreased in all subgroups, except for hypobaric females. Consequently, the influence of sex became highly significant (*p* < 0.001 for TDC and *p* < 0.01 for R), and the influence of hypoxia remained significant (*p* < 0.01 for TDC and *p* < 0.05 for R) due to the significant differences between hypobaric and control females, but not males.

Exploratory behavior in a hole board, measured as THV, was significantly influenced by hypoxia (*p* < 0.05), due to a significant difference between control and hypoxic males, but not females (Figure 4B). At adulthood, mean values decreased much more in males than in females, compared to the juvenile values, resulting in significantly higher exploratory behavior in females than in males (*p* < 0.001), while the influence of hypoxia was no longer significant. Anxiety-like behavior, measured in juvenile and adult rats as the percentage of the visited inner holes (% IN), did not significantly differ between the control and hypoxic rats or between males and females in either juvenile or adult animals (Figure 4B).

Social behavior was tested by measures of the amount of time that an animal spent exploring a novel object (TO) and exploring a conspecific (TR) (Figure 4C). TO was not influenced by hypoxia, sex, or their interaction at either juvenile or adult ages. Conversely, the influence of sex on TR was significant (*p* < 0.05). Interestingly, while control females explored a conspecific significantly longer than control males, the mean values of hypoxic males and females were very similar. At an adult age, mean values of TR increased in hypoxic animals of both sexes and in control males, in comparison to juvenile values, while they remained similar in control females. As a result, no significant effect of hypoxia, sex, or their interaction was observed in adult animals.

Spatial learning was tested separately for males and females. In juvenile females (Figure 4D left), only the testing day significantly influenced the number of correct choices (*p* < 0.0001), with significant improvements on day 3 vs. 2 and 5 vs. 4. In adult females, the number of correct choices still improved during testing days (*p* < 0.0001), but the learning curve was less steep than in juveniles, with significant improvements observed only on day 4 vs. 3. In juvenile males (Figure 4D right), both hypoxia (*p* < 0.05) and testing days (*p* < 0.0001) significantly influenced the number of correct choices, with control pups being overall more successful than hypoxic pups, and days 3, 4, and 5 bringing significant improvements in comparison to the corresponding previous day. In adult males, testing days still significantly influenced the number of correct choices (*p* < 0.0001), but the learning curve was also flattened, with more gradual improvement. While control rats still seemed to be more successful, the influence of hypoxia at an adult age became only indicative (*p* = 0.065).

### Chronic Structural Changes in the Mature Cingulate Cortex

**FIGURE 5:**
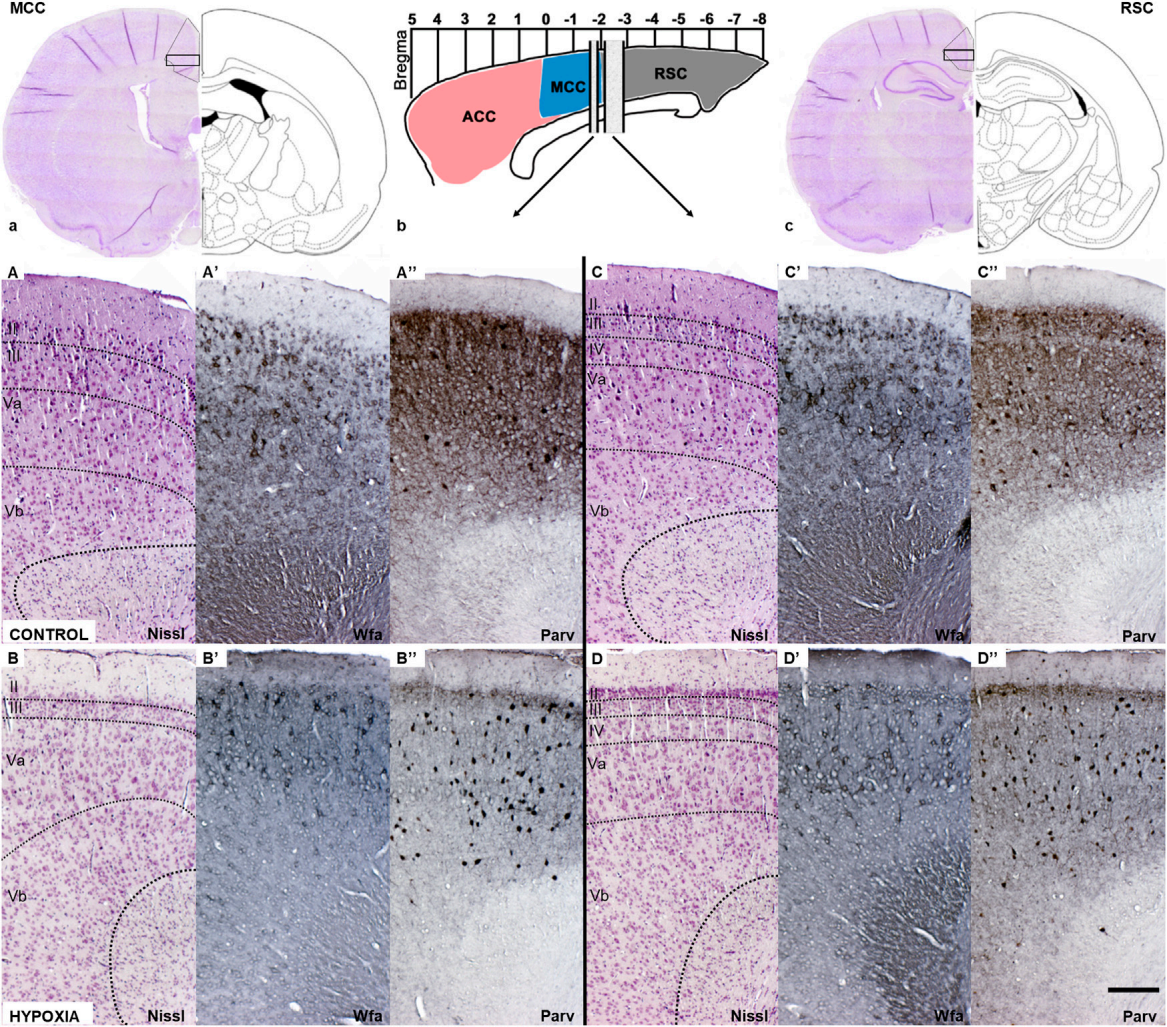
Coronal sections of the mature cingulate cortex at the age of 3.5 months indicate a downregulation of the diffuse Wfa-positive ECM and parvalbumin-positive neuropil, and concomitant upregulation of PNN expression around some neurons and parvalbumin-immunoreactivity of the interneurons’ soma in animals subjected to perinatal hypoxia. **(a)** Coronal section through cerebrum at the bregma level −1.56 mm display medial cingulate cortex area (MCC). The black rectangle frame indicates the part of the cortex presented below with higher magnification. **(b)** Schematic presentation of the position of ACC, MCC, and RSC regarding bregma scale and the position from which below presented sections are isolated. **(c)** Coronal section through cerebrum at the level of bregma −2.76 mm that displays retrosplenial cingulate cortex area (RSC). The black rectangle frame indicates the part of the cortex presented below with the higher magnification. **(A–D)** Coronal sections stained by cresylic-violet (Nissl modification) showing preserved gross morphology and proper cortical layering in the MCC and RSC in control and hypoxia-treated animals. **(A’–D’)** Coronal sections histochemically stained with Wisteria floribunda agglutinin (Wfa) show difference in qualitative characteristics of extracellular matrix where Wfa-positive matrix is diffusely present in all cortical layers and in the form of PNN around some neurons of predominantly cortical layers III and V. In contrast, in the hypoxia-treated animals the Wfa-positive extracellular matrix is localized almost exclusively in the form of PNN. The PNN are most frequently around interneurons of layers II/III and even more in layer V, especially prominent in RSC, in hypoxia treated animals compared to controls. **(A”–D”)** Coronal sections through MCC and RSC stained with parvalbumin-immunohistochemistry (Parv) indicate downregulated diffuse parvalbumin expression in the cortical neuropil of animals subjected to perinatal hypoxia. However, interneurons’ parvalbumin-positive soma were pronounced, especially in cortical layers II/III and V of these animals. Contrary, Parv-positive cingulate neuropil is pronounced in cortical layers II/III and V, but with less pronounced paralbumin expressing interneurons in controls. The actual image magnification from **(A–D”)**, is shown with the scale bar in **(D”)** presenting 200 µm.

The thorough anatomical examination of mature brains of adult rats and the histological examination of the stained sections showed preserved gross morphology (Figures 5a,c), the maintenance of regular cortical lamination (Figures 5A–D), and an absence of observable pathological features or consequences of the perinatal hypoxia.

**FIGURE 6:**
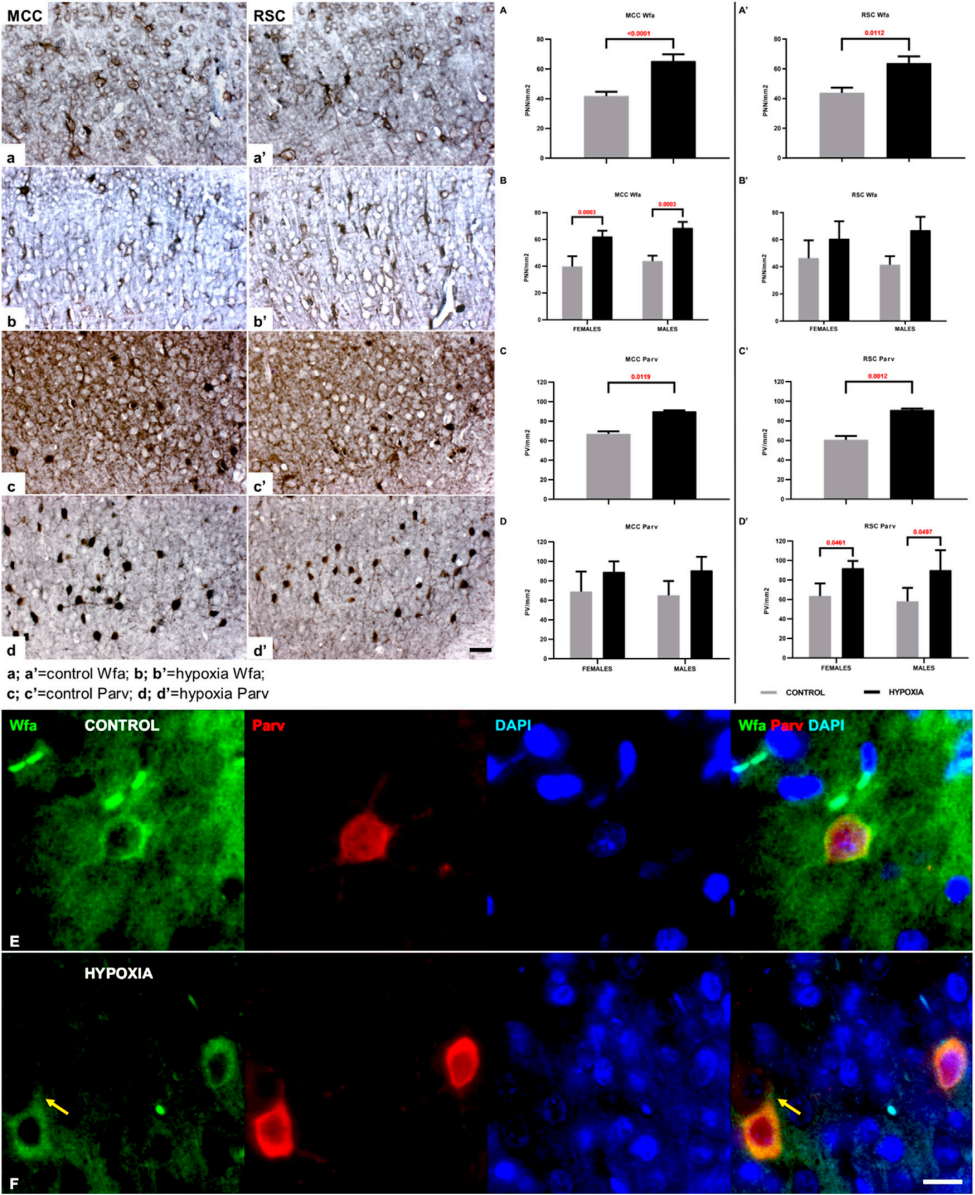
The qualitative and quantitative differences in perineuronal nets (PNN) and parvalbumin-positive interneurons (PV) between control and hypoxia-treated animals at the age of 3.5 months confirm a permanent structural change in the connectivity of the cingulate cortex. **(a–b’,A–B’)** High magnification of cortical layers III-Va of MCC **(a,b)**, cortical layers III-Va of RSC **(a’,b’)**, both stained by Wfa, and quantitative findings for the number of PNN in all cortical layers of MCC **(A,B)** and RSC **(A’,B’)**. The PNN are more numerous in hypoxia-treated animals in both cingulate areas. The Wfa staining of PNN is denser and thicker around the soma and proximal portion of the dendrites after perinatal hypoxia **(b,b’)** compared to controls **(a,a’)**. **(A–B’)** Two-way ANOVA revealed a significant influence of hypoxia on the number of PNN/mm2: **(A)** in the MCC (Hypoxia = F (1.12) = 72.46; *p* < 0.0001; Sex = F (1.12) = 3.641; *p* = 0.0806; Interaction = F (1.12) = 0.1863; *p* = 0.6736), and **(A’)** in the RSC area (Hypoxia = F (1.9) = 10.11; *p* = 0.0112; Sex = F (1.9) = 0.01477; *p* = 0.9059; Interaction = F (1.9) = 0.7814; *p* = 0.3997). The hypoxia-treated animals of both sexes had a significantly higher number of PNN in the MCC area **(B)**, and the same, but insignificant, trend was observed in RSC area **(B’)**. **(c–d’)** High magnification of layers III-Va of MCC **(c,c’)**, cortical layers III-Va of RSC **(d,d’)** stained by parvalbumin, and quantitative findings for the PV in all cortical layers of MCC **(C,D)** and RSC **(C’,D’)**. The interneurons soma is strongly parvalbumin - positively stained as well as the proximal portion of the dendrites in the animals subjected to perinatal hypoxia **(d,d’)** compared to controls **(c,c’)**. Two-way ANOVA revealed a significant influence of hypoxia on the number of PV per mm2: **(C)** in the MCC (Hypoxia = F (1.12) = 8.770; *p* = 0.0119; Sex = F (1.12) = 0.01956; *p* = 0.8911; Interaction = F (1.12) = 0.1141; *p* = 0.7413), and **(C’)** in the RSC area (Hypoxia = F (1.12) = 17.84; *p* = 0.0012; Sex = F (1.12) = 0.2751; *p* = 0.6095; Interaction = F (1.12) = 0.05901; *p* = 0.8122). The hypoxia-treated animals of both sexes had a significantly higher number of PV interneurons in the RSC area **(D’)**, while in the MCC area **(D)**, the difference is only indicative. Data are shown as mean ± standard error of the mean (SEM) (error bars). **(E)** A representative PNN (Wfa-positive, green) is visible around the cell body of PV (Parv, red) as a thin coat. The PV of the control animal also has larger soma when compared with PV in hypoxia-treated animal. **(F)** Representative PNNs (Wfa-positive, green) in the brain section of hypoxia-treated animal, show thicker and denser ECM-coat around the cell soma, surrounding the proximal portion of the dendrites, and often the initial axon segment [**(F)**; arrow]. The PV stained with parvalbumin (Parv, red) reveals the smaller neurons soma in the cingulate cortex of hypoxia-treated animal compared to control. The actual image magnification for **(a–d’)** is shown with the scale bar in **(d’)**, presenting 50 µm, for **(E,F)** is shown with the scale bar in **(F)**, presenting 25 µm.

A closer examination of two areas of the cingulate cortex (Figure 5, black rectangles in (a) and (c); and higher magnification A-D’’), the midcingulate are (MCC) (Figure 5A,A–B”) and the retrosplenial area (RSC) (Figures 5C,C–D”) disclosed differences in the extracellular matrix (ECM). A decrease of diffuse Wfa-positive ECM with a concomitant upregulation of PNN around a particular population of neurons (Figures 5A’–D’) was observable. The morphology of the individual PNN was also different due to more condensed and thicker Wfa staining around the soma, and proximal and basal dendrites, often including the axon-initial segment, in animals perinatally subjected to hypoxia (Figures 6a’,b’,Farrow). In the control animals, the PNN were thinner and primarily found around the cell bodies and proximal dendrites (Figures 5A’–D’, 6a,a’,E). In hypoxia-treated animals, the PNN were more numerous throughout the entire cortex, but especially in layers II/III and V (Figures 5A’–D’, 6a–b’). The tremendous significance of the impact of the hypoxia was observed in the increase of PNN number in the MCC area (*p* < 0.0001; Figure 6A) but was also highly statistically significant in the RSC (*p* = 0.0112; Figure 6A’). The effect of the perinatal hypoxia on the PNN number was notable in both sexes (Figures 6B,B’).

Likewise, indication of downregulated parvalbumin-positive neuropil was observed consistently in the sections from animals subjected to perinatal hypoxia (Figures 5A”–D”, 6c–d’). At the same time, an increase of the parvalbumin-immunoreactivity of the soma of interneurons was found in layers III and V (Figures 5A”-D”, 6c–d’). The qualitative examination also showed smaller cell soma of PV in hypoxia-treated animals. However, this parvalbumin-reactivity was pronounced in the soma and in the proximal portion of the dendrites in hypoxia-treated animals (Figures 6c–d’,E,F). The most remarkable difference between groups was observed in the number of PV in the MCC and the RSC (Figures 6C–D’). The quantification of PV confirmed the statistically significant effect of moderate perinatal hypoxia on PV numbers in both areas (*p* < 0.0119; Figure 6, C-D for MCC; *p* < 0.0012; Figures 6 C’,D’ for RSC). The effect of the hypoxia was notable for both sexes.

## Discussion

At this time, in developed countries, early and late premature babies’ survival rate is very high due to good perinatal medical care. However, perinatal events, most often hypoxia, still cause considerable deficits in these children, from mild to severe motor, neurological, cognitive, or behavioral disorders, which are more prominent in early premature babies. Despite all the available experimental methodologies, the longitudinal research into the relationship between molecular, histological, and behavioral changes after perinatal hypoxic injury is still highly challenging in humans and in animal models. The moderate forms of perinatal brain hypoxia, in particular, have not been extensively investigated in animal models. However, it could be a key to understanding the cause-consequence relationship of factors in the pathophysiology of several disorders and therefore essential for improvements in perinatal preventive and therapeutic strategies.

Briefly, this study disclosed the impact of moderate perinatal hypoxia on the intricate cingulate cortex connectivity and related behavioral features in a rat model. Moderate, single-event hypoxia in the rat neonates affected the proliferative zones and cell maturation in the acute phase that caused chronic, long-lasting effects such as the expansion of perineuronal nets and parvalbumin interneurons in the mid and retrosplenial cingulate cortex, and alteration of learning and behavioral patterns, for life.

Most previous studies have focused on combined HI injuries or severe hypoxia, which are often accompanied by excessive brain tissue inflammation, necrosis, gliosis, scarification, with periventricular leukomalacia followed by motor deficits as a dominant outcome (Millar et al., 2017). However, as designed, severe hypoxic or hypoxic-ischemic brain injury cannot model subtle changes and details of the phenomena of vulnerability and plasticity because of the much broader extent of lesion that affects many different cell populations and developmental processes simultaneously.

In contrast, we modeled moderate brain hypoxia that demonstrated cognitive and behavioral impairment in animals without motor deficits and significant brain pathology. The presented study is comparative and complementary to severe models showing less reactive microglia, hemorrhagic, or leukomalacia and so give better resolution in detecting affected developmental processes like proliferation, migration, axon elongation, synaptogenesis and ECM reorganization. Therefore, in this study, we investigated the immediate acute response to perinatal hypoxic injury, as the acid-base status, molecular (Hif-1α, Cox 4-1, Cas 3), cellular (microglia), and structural (SVZ) hallmarks, in the first step. Secondly, in the longitudinal follow-up study, we correlated this injury to long-term brain connectivity changes by examining cognitive and behavioral outcomes. Thirdly, we investigated the structural substrate in the brain for the found functional alteration. Finally, the study design provided proof of the concept of presumed high vulnerability to synaptogenesis demonstrated in a subpopulation of cells that influenced the formation of local neuron circuitries relevant to decision making, emotional learning, memory, and visual-spatial orientation functions.

### The Acute Molecular and Cellular Effect on Brain Development

The immediate effect of our moderate generalized hypoxia in rat pups was an increase of lactate in the blood of neonates. Increased lactate value in the circulation is a product of anaerobic metabolism in all high-oxygen-demanding tissues and thus brain tissue (Kraut and Madias, 2014). However, lactate is also considered a neuroprotective factor due to the neonatal brain’s high capacity to use lactate as an energy source. This assumption is based on two findings. The first one is the higher level of expression of monocarboxylate transporters and the concomitantly lower level of glucose transporters compared to the adult brain (Dumont et al., 2021; Roumes et al., 2021). The second finding is the metabolic cooperation called the astrocyte-neuron lactate shuttle, where the astrocytes fulfill neuronal energy needs using lactate (Pellerin and Magistretti, 2012). In that respect, the detected gradual increase of the Hif-1α expression in the brain, most notable 24 h after hypoxia, confirmed that a subset of brain cells was vulnerable, injured and responded to the hypoxic insult by an Hif-1α elevated expression. Still, the injury was not of such an extent to be statistically significant, as has been demonstrated in other studies (Semenza, 2000; Kaur et al., 2006). In addition, we found a statistically significant decrease of Cox 4-1 expression in hypoxia-treated animals 24 h after hypoxia. This finding supports previous claims of an increased Cox 4-1 proteolysis *via* the Hif-1α -induced mitochondrial protease activity found in hypoxic conditions (Fukuda et al., 2007). Jointly, these findings favor the idea of possible damage of oxidative processes in cells. Previously shown increases in Cas-3 activation, as early as 1 h post hypoxia (Wang et al., 2001), and lasting up to 6 days after injury, were shown as consequences of severe brain hypoxia and deterioration, which was not the case in our model. Thus, we may conclude that, in our non-invasive hypoxia model, the moderate brain injury could be confirmed by an acute, significant decrease of Cox 4-1, and an increase of Hif-1α as markers of impaired cell oxygenation. Still, the hypoxia itself was insufficient to cause enhanced developmental apoptosis, nor did it provoke “*de novo*” excessive cell death or any other pathological changes. The observed differences in the morphology of microglia between animals perinatally subjected to hypoxia and control animals in the cortex and the SVZ is further evidence supporting the presence of moderate injury. In our model, the morphology and number of the CD68 or Iba1 positive cells indicate mild microglial activation, likely provoked by enhanced developmental phagocytosis and remediation. It has been shown previously that, at P0, the majority of microglial cells in the white matter are rounded and amoeboid (as the microglia of the corpus callosum in our study); at P3, the cells develop broad stubby processes, which leads to the further development of processes at P7, and finally, a reduction in the number of microglia cells that occurs at P14 (Hristova et al., 2010). The microglial phagocytosis is fundamental for neural development (Fisch et al., 2020), with amoeboid microglia primarily present in the white matter, which was also observed (shown with the Iba1 immunostaining) in the corpus callosum of our model. The CD68 microglial cells located in the SVZ are largely phagocytic (Hristova et al., 2010; Shigemoto-Mogami et al., 2014; Fisch et al., 2020; Dumont et al., 2021), and, in our case, their increase suggests increased phagocytic activity associated with impaired proliferation and cell oxygenation, as evidenced by lower SVZ cell density. The ramified Iba1 positive microglia cells with processes that extended the diameter of the microglial soma more than two-fold, were observed within the CG, and in the SVZ 24 h after subjection to hypoxia, indicating precocious maturation of some microglial cells, similar to findings reported in periventricular white matter injury (Hristova et al., 2010).

### The Long-Lasting Signature of Perinatal Hypoxia on Cortical Connectivity and Behavior

The ECM has been shown to play an important role in brain development, being one of the major or key building constituents of transient fetal brain structures (such as preplate, subplate, marginal zone, crossroads of axonal pathways, perinatally remnants of subplate) that precede the establishment of cortical lamination and connectivity in the human brain (Kostović et al., 2014a, 2014b; Milošević et al., 2014; Milos et al., 2020). The ECM is the fourth constituent of the quadripartite synapse (presynaptic, postsynaptic, glial and ECM elements; reviewed in Sykova and Nicholson, 2008). The specialized condensed ECM known as PNN are critical in synaptogenesis, synapse maturation and maintenance, and ultimately, in plasticity after injury (for reviews, see Kwok et al., 2014; Sorg et al., 2016; Nalivaeva et al., 2018; Shen, 2018; Duncan et al., 2019; Fawcett et al., 2019; Van’t Spijker et al., 2019; Carulli and Verhaagen, 2021). Several studies have investigated specifically, the changes in PNN after severe HI in adult animals and have shown that HI injury causes decomposition of PNN in infarcted neocortical areas (Härtig et al., 2017; Wen et al., 2018). The experience-dependent synaptic plasticity in adulthood relies particularly on PNN around fast-spiking PV within many brain regions (Wang and Fawcett, 2012; Sorg et al., 2016). The PNN show region-specific features (Ueno et al., 2017a; Ueno et al., 2017b; Ueno et al., 2018) and vulnerability, as evidenced in a variety of neurological and psychiatric disorders (Nalivaeva et al., 2018; Wen et al., 2018; Fawcett et al., 2019; Carulli and Verhaagen, 2021). We were interested to see whether moderate perinatal hypoxia executed before the complete differentiation of the ECM, PNN, and PV, still affects PNN formation, number, and/or distribution, regardless of whether it is a consequence of injury or plasticity response. Determining whether these changes in ECM organization in the brains of animals that underwent perinatal hypoxia could lead to altered behavior or learning abilities later, in adolescence or adulthood, was of no less interest and importance. It would be also interesting to test in the future a life-long effect of perinatally occurring hypoxia, possible alterations of PV and PNN connectivity and behavior since astrocyte and microglia activity were shown up to 2 years after ischemic reperfusion insult (Sekeljic et al., 2012; Radenovic et al., 2020).

This study disclosed qualitative differences in the morphology and distribution of PNN and PV, with a significant increase in the density of PNN and PV in the hypoxia-treated animals of both sexes, in two areas of interest: the MCC and RSC. Other studies have also shown an increased number of PNN after: sleep deprivation and oxidative stress in PV neurons (Harkness et al., 2019); acquisition of auditory fear memory in the hippocampus, auditory cortices, and anterior cingulate cortex (ACC) (Banerjee et al., 2017; Shi et al., 2019); formation of cocaine-related preference memories [around cerebellar Golgi neurons (Carbo-Gas et al., 2017)]; and after repeated cocaine administration (Slaker et al., 2018). The PNN protect neurons from oxidative stress, as shown in several studies (Cabungcal et al., 2013; Suttkus et al., 2014), but simultaneously they are susceptible to such stress, as well (Cabungcal et al., 2013). The enzymatic removal of PNN generally increases the reverse-learning required for behavioral flexibility (Happel et al., 2014). In the cerebellar nuclei, enzymatic removal of PNN results in better motor-associative learning (Carulli et al., 2020). In the hippocampus, the removal of PNN reverses learning deficits (Bertocchi et al., 2021). Since we found changes in the number of PNN in the MCC and the RSC, it is worth mentioning that the putative role of MCC is to connect the ACC and the RSC in relevant learning by the selection of different learning pathways and patterns. The MCC also is important in decision-making and emotional learning (Vann and Aggleton, 2005; Lukoyanov and Lukoyanova, 2006; Vogt and Paxinos, 2014). Spatial navigation, learning of reward-based tasks, regulation of emotional responsiveness to new situations, as well as memory, and learning by visual cues are attributed to the RSC (Cooper and Mizumori, 2001; Vann and Aggleton, 2005; Lukoyanov and Lukoyanova, 2006; Vogt and Paxinos, 2014). In our model, hypoxia affected two of the three main aspects of novel space exploration. Locomotor activity was significantly affected in juveniles of both sexes and adult females. Exploratory behavior was significantly affected in juvenile males, while thigmotaxic behavior was not affected. That implies that affected connectivity after neonatal hypoxia induces long-lasting hyperactivity without affecting anxiety level. Most studies used either one sex or both sexes in a single group, preventing the identification of possible sex-specific vulnerability to hypoxia. In contrast, this study showed that significant hyperactivity was transitory for males, while females remained equally hyperactive in adulthood, suggesting higher vulnerability. Differential male/female vulnerability to hypoxia found in the social-choice test, which showed that hypoxia attenuated sex differences in sociability that initially existed, was additional supporting evidence. Previous studies also report hyperactivity measured as an increase in ambulation and/or rearing, in juvenile rats after exposure to intermittent postnatal hypoxia at days P7-10 (Decker et al., 2003; Decker et al., 2005), or mild chronic hypoxia at P10 (Mikati et al., 2005) and to neonatal anoxia (Shimomura and Ohta, 1988; Buwalda et al., 1995; Iuvone et al., 1996). In addition to transitory hyperactivity (Shimomura and Ohta, 1988; Iuvone et al., 1996), long-lasting learning impairments have been considered one of the hallmarks of hypoxia in young (Raveendran and Skaria, 2012) and adult rats (Buwalda et al., 1995; Iuvone et al., 1996; Decker et al., 2003; Mikati et al., 2005; Galeano et al., 2014), as was also confirmed in our study for moderate perinatal hypoxia. Although more studies are required before drawing a firm conclusion, we can assume that the increase in PNN and PV could have been responsible for limited synaptic plasticity in the cingulate cortex, consequent hyperactivity, and restriction of cognitive functions in our model. In other words, developmentally altered PNN-synapse formation that is associated with a specific neuron population, in fact, highlights lesions that affect connectivity function, as evidenced by behavior alterations. In its more pronounced form, developmentally disturbed PNN-related connectivity may account for cognitive or behavioral disorders, such as schizophrenia, bipolar disorder, major depression, or the autism spectrum. Developmentally unfavorable conditions such as hypoxia during midgestation in the human or neonatal rats may also reduce the electric activity of the early microcircuits in the subplate zone (for review, Molnar et al., 2020). Therefore, a further research focus in this model may be the vulnerability of sublate and/or layer 6b neurons. The lesions of these neurons affect the development of thalamocortical and corticothalamic connections that may be the substrate of subsequent challenging conditions as cognitive, psychiatric, or neurological disorders (McQuillen et al., 2003; Sheikh et al., 2019; Molnar et al., 2020).

Our model may have comparative and complementary advantages and potential in researching the pathogenesis of these disorders of a neurodevelopmental origin.

## Conclusion

The unique study model of perinatal brain injury enabled the assessment of behavioral and learning alterations and subsequent detection of related changes in the quality and number of PNN and PV after moderate non-invasive hypoxia. This research confirms the resistance of the immature brain to hypoxia. However, when excessive, even a single perinatal hypoxic event disturbs the proliferation in the SVZ and the ongoing organization of the ECM relevant for the synaptogenesis, synapse maturation, and synaptic plasticity in interneurons. A very plausible consequence of such an event might be a detrimental effect on cortical circuitries and connectivity; thus, increased cognitive vulnerability and altered neural functions that become manifest under demanding conditions or later in life.

As such, this research design provides a better understanding of the brain’s cellular and extracellular substrate of vulnerability to perinatal hypoxic injury and the relationship to long-term behavioral and cognitive outcomes. In addition, this model shows excellent potential for research into predisposing factors accountable for neurological or neuropsychiatric conditions. Thus, the perinatal vulnerability of synaptogenesis seems to be the basis for altered structural and behavioral features later in life. Therefore, we believe this model is most suitable for research into neurological and psychiatric conditions of developmental (fetal or early postnatal) origin. Further research in comparative and translational studies on this model in rats is expected to provide new insights into possible prevention strategies and therapeutic targets to alleviate or treat the consequences of developmental brain lesions in humans.

## Materials and Methods

All animal experiments comply with the ARRIVE guidelines and have been carried out following the United Kingdom. Animals (Scientific Procedures) Act, 1986, EU Directive 2010/63/EU, Croatian regulations, and associated documents (NN 102/2017 and 32/19; NN 55/2013 and 39/17) for experimentations on animals. The study design and experiments were approved by the Ethical Committee of the University of Zagreb and national Ethical and Animal welfare bodies (EP231/2019; UP/I-322-01/19.01/75). Every effort was made to reduce the number of animals in use and to minimize animal discomfort. The sex of animals is indicated, and where appropriate, the influence (or association) of sex on the study results. The minimum number of needed animals (82) was determined by power analysis, and animals were randomly assigned to the hypoxic or control group, always maintaining equal sex representation. The timeline of the study design is presented in Figure 1 (Figure 1A). All methods used in the study are described in detail in the Supplementary Material.

### Experimental Subjection to Hypoxia

The Wistar Han (RccHan: WIST) rat pups at postnatal day 1 (P1) were weighed, sex determined and marked by a permanent toe tattoo (NEO-9 Neonate Tattoo System, AgnTho’s AB, Sweden). The day of birth is considered P0 until the noon of the next day after which P1 starts. The hypoxic group (3F+3M per session) was placed in a hypoxic chamber (diameter of chamber: 300 mm; volume of chamber: 12,9L) with litter bedding, at 27°C, with a partial pressure of oxygen (pO_2_) of 73 mmHg and an atmosphere pressure (p^ATM^) of 350 mmHg, for 2 h. The control group (3F+3M per session) was exposed to a pO_2_ of 159 mmHg and a p^ATM^ of 760 mmHg, with other parameters the same as the hypoxic group. The average weight of animals used for experimental purposes was 6.88g +/− 10%. The hypoxia model is designed by modifying the previously described protocol (Kaur et al., 2006). After the procedure, the rat pups were used for acid-base status measurement or were returned to their dams and used later in the study (see timeline in Figure 1A).

### Acid-Base Status Measurement

Immediately after the experimental hypoxia, six pups per group were decapitated. The blood was collected in mini capillary tubes (75 µL per animal, separately) for instant analysis of the acid-base status using i-STAT Alinity gas analyzer (Abbott, CG4+ test Cartridge). The pH (hydrogen potential), pCO2 (partial pressure of carbon dioxide), pO2 (partial pressure of oxygen), BE, ecf (base excess in the extracellular fluid), HCO_3_^−^ (bicarbonate), sO2 (oxygen saturation), TCO2 (total carbon dioxide), and lactate were measured in all blood samples.

### Western Blot, Histology, Immunohistochemical Methods, and Quantification

For the Western blot method (WB), six pups per group were anesthetized by body cooling [+4°C/2 min; (Phifer and Terry, 1986)] and decapitated 2, 8, or 24 h after subjection to hypoxia, and the brains were snap-frozen. Primary and secondary antibodies used for protein detection in WB and immunohistochemistry are listed in Table 1. The WB and protein analysis were performed as described previously (Herrera-Molina et al., 2017).

**TABLE 1:** List of primary and secondary antibodies used in Western blot and immunohistochemistry.

Primary antibodies (clone)	Cat. No.	Host, isotype, format	Dilution	Supplier
Hif 1 alpha antibody (H1alpha67)	NB100-105	Mouse monoclonal	1:500	Novus biologicals, Centennial, Colorado, US
		Protein G purified		
Cox 4-1	AF5814	Goat polyclonal unconjugated	1:200	R&D systems Minneapolis, Minnesota, US
Cleaved caspase-3 (Asp175)	9661	Rabbit polyclonal	1:1,000	Cell signaling, Leiden, Netherlands
Microglia marker anti Iba-1	019-19741	Rabbit	1:1,000	FujiFilm wako chemicals, US
CD68	MCA341R	Mouse monoclonal purified	1:200	Biorad, Hercules, California US
Anti-parvalbumin antibody	ab11427	Rabbit polyclonal unconjugated	1:2,000	Abcam, Cambridge, United Kingdom
Biotinylated wisteria floribunda agglutinin (Bio-Wfa)	L1516	—	diluted 6 µg on 1 ml	Sigma—Aldrich, Saint Louis, US
Fluorescein wisteria floribunda lectin (Wfa, Wfl)	FL-1351	—	diluted 5 µg on 1 ml	Vector laboratories, Inc., Burlingame, US
Secondary antibodies	Cat. No.	Host, isotype, format	Dilution	Supplier
Peroxidase-affiniPure secondary antibody	705-035-003	Donkey anti-goat IgG (H + L)	1:50,000	Jackson immunoresearch, Ely, United Kingdom
			1:200	
Peroxidase-affiniPure secondary antibody	711-035-152	Donkey anti-rabbit IgG (H + L)	1:50,000	Jackson immunoresearch, Ely, United Kingdom
			1:200	
Alexa fluor 488, highly cross-adsorbed secondary antibody	A21206	Donkey anti-rabbit IgG (H + L)	1:1,000	Thermo fisher Scientific, Waltham, Massachusetts, US
Alexa fluor 546, highly cross-adsorbed secondary antibody	A10040	Donkey Anti-Rabbit IgG (H + L)	1:1,000	Thermo fisher scientific, Waltham, Massachusetts, US
Alexa fluor 647, highly cross-adsorbed secondary antibody	A21447	Donkey Anti-Goat IgG (H + L)	1:1,000	Thermo fisher scientific, Waltham, Massachusetts, US

The brain structure was analyzed on coronal sections, cryo-sections 60 µm thick for P1, and paraffin 14 µm thick for P105, stained by cresyl-violet (modification by Nissl: 0.5% Cresyl violet, Chemika, Girraween, NSW, Australia). The differential expression of specific proteins was examined by immunofluorescent (IF; free-floating 60 μm thick cryo-sections for P1; glass-mounted 14 µm thick sections for P105) and classic non-fluorescent (IHC) (free-floating 60 μm thick cryo-sections for P1; glass-mounted 14 µm thick sections for P105) staining as described in our previous publications (Bobić Rasonja et al., 2019; Culjat and Milošević, 2019), and given in more details in the Supplementary Material.

The number of PNN and parvalbumine-positive interneurons (PV) were counted in the cingulate cortex at the levels of the bregma −1.56 mm to bregma −1.92 mm for the midcingulate area (MCC), and bregma −2.04 mm to bregma −2.92 mm for the retrosplenial area (RSC) (Paxinos and Watson, 2007). The quantitative analysis (eight animals per group) was performed using Neurolucida 10 (MBF–Bioscience, Williston, ND, United States), and an Olympus BX61 microscope as described previously (Bicanic et al., 2017).

### Behavioral Testing

Forty rats were submitted to open-field, hole-board, T-maze, and social choice tests as described previously (Blazevic et al., 2012). Briefly, horizontal locomotor activity (total distance covered in cm, TDC), and the number of rearing times (R) were tested in the open field for 5 minutes. Exploratory and thigmotactic behaviors were tested in a hole-board as the total number of holes (THV), and the percentage of the inner holes (% IN) visited for 5 minutes. Learning was tested in a T-maze as the number of correct choices in 10 consecutive trials during five successive days. Sociability was tested as the amount of time spent exploring an inanimate object (TO, in seconds) and a conspecific (TR, in seconds) for 5 minutes. Rats were tested according to the same protocol at P30 and P70.

### Statistical Analysis

All statistical tests were conducted using Prism8 (GraphPad Software, Inc., La Jolla, CA, United States) and JMP 11.2 (SAS Institute Inc., Cary, NC, United States). Due to the small number of samples, it was not possible to reliably prove the normality of the distribution for analysis of the acid-base status, so we decided to use an unpaired, two-tailed, nonparametric Mann-Whitney test. An unpaired, two-tailed, Student’s t-test was used to examine the difference between groups in protein quantity on Western blots. An independent two-way ANOVA was used to check for the influences of hypoxia and gender on behavioral parameters and on the number of PNN and PV. The univariate split-plot approach for repeated measures ANOVA was used to analyze the influences of experimental hypoxia (between-subject variable) and testing day (within-subject variable) on the number of correct choices in the T-maze test. Tukey’s honest significance test was used for post-hoc analyses. The level of significance was set to 0.05 (two-tailed *p* value). Values in the text were expressed as mean ± standard error of the mean (SEM).

## Data Availability

The original contributions presented in the study are included in the article/Supplementary Material, further inquiries can be directed to the corresponding author.

## Glossary

ACC: anterior cingulate cortex (area)
A-B: acid-base status
BE, ecf: base excess in the extracellular fluid
Cas 3: cleaved caspase-3
CC: the number of correct choices in T-maze test
CD68: class D scavenger receptor 68
CG: cingulate gyrus
Cox 4-1: cytochrome-c-oxidase subunit-4 isoform-1
DAPI: 4′,6-diamidino-2-phenylindole; a blue-fluorescent DNA stain
ECM: extracellular matrix
F: female
HCO_3_^−^: bicarbonate
HI: hypoxic-ischemic
Hif-1α: hypoxia-inducible factor-1-alpha
Iba-1: ionized-calcium-binding-adaptor-molecule-1
IF: immunofluorescence
IHC: immunohistochemistry
M: male
MCC: mid-cingulate cortex (area)
Parv: parvalbumin
p^ATM^: atmosphere pressure
PBS: phosphate-buffered saline
FA: formaldehyde
pH: hydrogen potential
PNN: perineuronal nets
PV: parvalbumin-expressing interneurons
P: postnatal day
R: number of rearings
RSC: retro-splenial cortex (area)
RT: room temperature
SEM: mean ± standard error of the mean
SVZ: subventricular zone
TDC: total distance covered in cm
THV: total number of holes visited
TO: time spent exploring an inanimate object
TR: time spent exploring a conspecific
WB: Western blot method
Wfa: Wisteria floribunda agglutinin
% IN: the percentage of the inner holes visited
