# Supplementary material for: The Signature of Moderate Perinatal Hypoxia on Cortical Organization and Behavior: Altered PNN-Parvalbumin Interneuron Connectivity of the Cingulate Circuitries
Source: Front Cell Dev Biol. 2022 Feb 28;10:810980. doi: 10.3389/fcell.2022.810980 (PMC8919082; doi:10.3389/fcell.2022.810980)
Supplement: Supplementary file 1 [file Table1.pdf]

Supplementary Table 1. The total number of animals used in the study.

EXPERIMENT	AGE (when sampled)	TOTAL NUMBER OF ANIMALS	HYPOXIA-TREATED ANIMALS	CONTROL ANIMALS
ACID-BASE STATUS RECORDING	P1	12 (6F; 6M)	6 (3F; 3M)	6 (3F; 3M)
WESTERN-BLOT PROTEIN DETECTION AND ANALYSIS	P1	18 (9F; 9M)	9 (5F; 4M)	9 (4F; 5M)
	2h after hypoxia	6 (3F; 3M)	3 (1F; 2M)	3 (2F; 1M)
	8h after hypoxia	6 (3F; 3M)	3 (2F; 1M)	3 (1F; 2M)
	24h after hypoxia	6 (3F; 3M)	3 (2F; 1M)	3 (1F; 2M)
IMMUNOHISTOCHEMICAL EXPERIMENTS AND ANALYSIS	P1	12 (6F; 6M)	6 (3F; 3M)	6 (3F; 3M)
	8h after hypoxia	6 (3F; 3M)	3 (1F; 2M)	3 (2F; 1M)
	24h after hypoxia	6 (3F; 3M)	3 (2F; 1M)	3 (1F; 2M)
	P105	30 (16F; 14M)	16 (9F; 7M)	14 (7F; 7M)
BEHAVIORAL TESTING	P30 AND P70	40 (20F; 20M)	22 (10F; 12M)	18 (10F; 8M)

P – postnatal day, F - female, M – male.
