# Supplementary material for: The Signature of Moderate Perinatal Hypoxia on Cortical Organization and Behavior: Altered PNN-Parvalbumin Interneuron Connectivity of the Cingulate Circuitries
Source: Front Cell Dev Biol. 2022 Feb 28;10:810980. doi: 10.3389/fcell.2022.810980 (PMC8919082; doi:10.3389/fcell.2022.810980)
Supplement: Supplementary file 2 [file Table2.pdf]

Supplementary Table 2. Activity, exploratory and social behavior of control and hypoxic rats at juvenile and adult age.

Test	Parameter	Sex	JUVENILE RATS			ADULT RATS		
			Treatment			Treatment		Sex mean
			control	hypoxic	Sex mean	control	hypoxic	
Open field	TDC (cm)	male	1253 ± 222	1825 ± 190	**1584 ± 146**	577 ± 185	682 ± 151	**634 ± 119*** **
		female	1593 ± 199	2023 ± 199	**1808 ± 141**	1013 ± 165&	1835 ± 165	**1424 ± 117**
		**Treatment mean**	**1442 ± 149#**	**1919 ± 137**		**819 ± 124##**	**1206 ± 112**	
	R	male	8.63 ± 2.26	15.2 ± 1.85	**11.9 ± 1.46**	4.50 ± 1.91	5.58 ± 1.28	**5.15 ± 1.39** **
		female	11.9 ± 2.02	15.9 ± 2.02	**13.9 ± 1.43**	6.60 ± 1.64&	14.9 ± 2.72	**10.8 ± 1.36**
		**Treatment mean**	**10.4 ± 1.52#**	**15.5 ± 1.37**		**5.67 ± 1.44#**	**9.82 ± 1.30**	
Hole board	THV	male	19.0 ± 3.41$	33.3 ± 2.61	**26.2 ± 2.15**	8.25 ± 1.44	8.25 ± 1.71	**8.25 ± 1.73*** **
		female	28.0 ± 3.01	27.1 ± 2.86	**27.6 ± 2.08**	16.9 ± 3.57	17.9 ± 2.13	**17.4 ± 1.69**
		**Treatment mean**	**23.5 ± 2.28#**	**30.2 ± 1.93**		**13.1 ± 1.79**	**12.6 ± 1.62**	
	%IN	male	10.4 ± 3.77	10.1 ±1.69	**10.2 ± 1.88**	23.6 ± 3.40	15.5 ± 6.13	**18.9 ± 3.07**
		female	12.0 ± 3.48	8.79 ± 1.30	**10.5 ± 1.90**	22.9 ± 3.49	19.3 ± 1.61	**21.1 ± 2.95**
		**Treatment mean**	**11.3 ± 1.96**	**9.53 ± 1.82**		**23.2 ± 3.13**	**17.36 ± 2.89**	
Social choice	TO (s)	male	12.6 ± 3.56	5.36 ± 3.03	**8.42 ± 2.48**	14.1 ± 3.72	13.2 ± 2.97	**13.6 ± 2.26**
		female	10.5 ± 3.18	7.86 ± 3.80	**9.41 ± 2.08**	8.78 ± 3.28	13.7 ± 3.11	**11.4 ± 2.38**
		**Treatment mean**	**11.4 ± 2.39**	**6.33 ± 2.43**		**11.1 ± 2.48**	**13.4 ± 2.15**	
	TR (s)	male	42.3 ± 13.9@	44.7 ± 11.8	**43.7 ± 9.10***	87.4 ± 13.3	93.0 ± 11.34	**90.6 ± 8.74**
		female	95.8 ± 12.4	49.3 ± 14.8	**76.6 ± 9.65**	96.4 ± 11.9	74.8 ± 11.9	**85.6 ± 8.74**
		**Treatment mean**	**72.0 ± 9.29**	**46.5 ± 9.47**		**92.4 ± 8.92**	**17.36 ± 2.89**	

# p<0.05, ##p<0.01 control vs. hypoxic, Two-way ANOVA; *p<0.05, **p<0.01, ***p<0.001, male vs. female, Two-way ANOVA; $p<0.05, control males vs. hypoxic males, & p<0.05, control females vs. hypoxic females, @p<0.05 control males vs. control females, Tukey's HS post-test after Two-way ANOVA. Values are shown as mean ± standard error. TDC – Total Distance Covered and R- number of rearings in an open field; THV – Total number of Holes Visited and %IN – percentage of inner holes in a hole board; TO – time spent exploring an object and TR – time spent exploring a conspecific in social choice test.
