# Supplementary material for: The Signature of Moderate Perinatal Hypoxia on Cortical Organization and Behavior: Altered PNN-Parvalbumin Interneuron Connectivity of the Cingulate Circuitries
Source: Front Cell Dev Biol. 2022 Feb 28;10:810980. doi: 10.3389/fcell.2022.810980 (PMC8919082; doi:10.3389/fcell.2022.810980)
Supplement: Supplementary file 3 [file Table3.pdf]

Supplementary Table 3. Parameters of two-way ANOVA analysis of behavior in open-field, hole-board and social-choice tests, and of repeated-measure ANOVA of behavior in a T-maze test.

Test	Parameter	JUVENILE RATS Treatment	Sex	Interaction	ADULT RATS Treatment	Sex	Interaction
Open-field	TDC	F(1, 35)=6.11 p=0.018	F(1, 35)=1.76 p=0.193	F(1, 35)=0.12 p=0.728	F(1, 36)=7.69 p=0.009	F(1, 36)=22.6 p<0.0001	F(1, 36)=4.60 p=0.039
	R	F(1, 36)=6.65 p=0.014	F(1, 36)=0.96 p=0.333	F(1, 36)=0.39 p=0.538	F(1, 35)=5.84 p=0.021	F(1, 35)=8.65 p=0.006	F(1, 35)=3.46 p=0.071
Hole-board	THV	F(1, 34)=5.06 p=0.031	F(1, 34)=0.22 p=0.646	F(1, 34)=6.51 p=0.015	F(1, 33)=0.04 p=0.837	F(1, 33)=14.35 p=0.0006	F(1, 33)=0.04 p=0.837
	%IN	F(1, 35)=0.419 p=0.522	F(1, 35)=0.003 p=0.955	F(1, 35)=0.297 p=0.589	F(1, 34)=1.88 p=0.179	F(1, 34)=0.128 p=0.722	F(1, 34)=0.239 p=0.598
Social-choice	TO	F(1, 32)=2.11 p=0.156	F(1, 32)=0.003 p=0.957	F(1, 32)=0.46 p=0.503	F(1, 33)=0.36 p=0.551	F(1, 33)=0.54 p=0.466	F(1, 33)=0.80 p=0.377
	TR	F(1, 32)=2.75 p=0.107	F(1, 32)=4.80 p=0.036	F(1, 32)=3.41 p=0.074	F(1, 35)=0.43 p=0.515	F(1, 35)=0.14 p=0.708	F(1, 35)=1.26 p=0.270
T-maze	CC	**Treatment**	**Time**	**Interaction**	**Treatment**	**Time**	**Interaction**
	males	F(1, 15.7)=5.37 p=0.034	F(4, 74)=65.6 p<0.0001	F(4,74)=0.27 p=0.896	F(1, 15.2)=3.97 p=0.065	F(4, 73.7)=18.0 p<0.0001	F(4, 73.7)=0.44 p=0.781
	females	F(1, 17.9)=0.28 p=0.601	F(4, 94.4)=27.4 p<0.0001	F(4, 94.4)=0.49 p=0.743	F(1, 19)=0.43 p=0.519	F(4, 76)=18.9 p<0.0001	F(4, 76)=1.09 p=0.369

TDC – Total Distance Covered; R- number of rearings; THV – Total number of holes visited; %IN – a percentage of inner holes visited; TO – time spent exploring an object; TR – time spent exploring a conspecific; CC – number of correct choices.
