# Supplementary material for: The Signature of Moderate Perinatal Hypoxia on Cortical Organization and Behavior: Altered PNN-Parvalbumin Interneuron Connectivity of the Cingulate Circuitries
Source: Front Cell Dev Biol. 2022 Feb 28;10:810980. doi: 10.3389/fcell.2022.810980 (PMC8919082; doi:10.3389/fcell.2022.810980)
Supplement: Supplementary file 4 [file DataSheet1.docx]

Supplementary Material

**Material and Methods**

Animal housing and study design

Animals were housed in polysulfone cages in a controlled temperature (21 ± 2°C) and humidity (65 ± 5%) environment. A light/dark cycle of 12:12h was employed with the light period beginning at 7 a.m. Food (4RF21C, Mucedola srl, Settimo Milanese MI, Italy) and tap water were provided *ad libitum*. The minimum number of animals needed for the study was determined by power analysis and a total of 82 one-day-old Wistar Han (RccHan:WIST) rats were obtained from our breeding facility (School of Medicine, University of Zagreb, Croatia). The day of birth is considered P0 until the noon of the next day, when P1 begins. Pups (without any dysmorphic features of an average body mass 6.8g +/-10%) were randomly assigned to the hypoxic or control group keeping both sexes equally represented per experimental group. The numbers of animals used per experiment in this research are described in Supplementary Table 1.

Sampling, tissue and protein isolation, and Western blot method

Eighteen animals (8F; 10M) from hypoxic (n=10; 5F; 5M) and control (n=8; 3F and 5M) group were sacrificed 2h, 8h, and 24h after hypoxic treatment. Rats were anesthetized by sub-cooling on ice to 4°C using an ice-filled substrate covered with cellulose for up to 2 min (Phifer and Terry, 1986) (https://www.bu.edu/researchsupport/compliance/animal-care/working-with-animals/anesthesia/anesthesia-and-analgesia-neonatal-mice-and-rats-iacuc/) and then decapitated. Brains were isolated, snap frozen in liquid nitrogen and stored at -80˚C.

Protein isolation

Total brain proteins were isolated as follows: brains were homogenized in RIPA buffer (50mM Tris HCl, pH 7.4, 150 mM NaCl, 1% Triton X-100, 0.5% sodium deoxylcholate, 0.1% SDS, 1mM PMF, 5mM NaF, 1mM Na3VO4) with protease inhibitors cocktail (Roche, Basel, Switzerland‎ #11836153001) using Potter-Elvhjem homogenizer and teflon pestle. Samples were centrifuged at 12 000 x g for 20 min/4˚C. Supernatant was used for protein analysis.

Western blot method (WB)

Proteins were fractionated by SDS-PAGE (NuPage 4-12% BisTris gels #NP0322, in NuPAGE™ MOPS SDS Running Buffer #NP0001) and transferred to a 0.45μm polyvinylidene difluoride membrane (Thermo Fisher, Waltham, Massachusetts, USA #88518) in NuPAGE™ Transfer Buffer #NP0006 using a transfer apparatus according to the manufacturer’s protocols (Invitrogen by Thermo Fisher, Waltham, Massachusetts, USA #88518). After transfer, membranes were incubated with No-Stain protein Labeling reagent (#A44449) according to manufacturer’s instructions and total proteins were visualized using ChemiDoc XRS; imager (Bio-Rad, Hercules, California, USA #88518 #1708265) (Supplementary Figure 1). After incubation with 5% nonfat milk in PBST (137mM NaCl, 2.7mM KCl, 8mM Na2HPO4, 2mM KH2PO4, 0.1% Tween-20) for 60 min, the membranes were incubated with primary antibody at 4˚C overnight (Li et al., 2006; Chen et al., 2008; Deng et al., 2019). Primary and secondary antibodies used in WB are listed in Table 1. For chemo-luminescent signal detection SuperSignal West Femto Maximum Sensitivity Substrate (Thermo Scientific #34096 Lot #SB244813; Rivero-Gutiérrez et al., 2014) was used. We used total protein signal on the membranes and chemo-luminescent signal of proteins for protein quantification in Image Lab software according to manufacturer’s instructions (Bio Rad).

Sampling, tissue and sections preparation for histology and immunohistochemistry

For histology and immunohistochemical methods on frozen sections in purpose to analyze acute changes in the brain after hypoxic treatment, 12 animals (6F and 6M) from hypoxic (n=6; 3F and 3M) and control (n=6; 3F and 3M) group were sacrificed at 8h, and 24h after hypoxic treatment (Supplementary Table 1). P1 rats were anesthetized by sub-cooling on ice to 4°C using an ice-filled substrate covered with cellulose for up to 2 min (Phifer and Terry, 1986) (https://www.bu.edu/researchsupport/compliance/animal-care/working-with-animals/anesthesia/anesthesia-and-analgesia-neonatal-mice-and-rats-iacuc/). After making sure that the pups are under deep anesthesia (blue-purple color of the skin, stops breathing spontaneously at regular intervals and stops moving), rats were transcordially perfused (Gage et al., 2012) with 10 ml ice-cold phosphate-buffered saline (PBS), subsequently with 10 ml 4% formaldehyde (FA, 0.1 M phosphate buffer; pH = 7.4) and decapitated. Brains were isolated and post-fixed for 18h in 4%FA/ 4°C and cryo-protected by immersion (gradually) to 30% sucrose solution during 48h/4°C. The brains were rapidly frozen in isopentane at -80°C. To cut sections, brains were attached with O.C.T. Compound (Tissue-Tek; Sakura Finetek, Europe B.V., NL) and then cut in coronal 60-μm thick sections (bregma 0,40mm bregma -1,60mm) (Ramachandra and Subramanian, 2011) using a cryostat (CM1950; Leica Biosystems, USA) at -25°C. Brain sections containing regions of interest were collected in ice-cold PBS containing 0.05% sodium-azide. For analysis of chronic changes in the brain after hypoxic treatment 30 animals (16F and 14M) from hypoxic (n=16; 9F and 7M) and control (n=14; 7F and 7M) group were sacrificed 3.5 months after hypoxic treatment. Rats were deeply anesthetized with intraperitoneal administration mixture of 80 mg/kg ketamine and 10 mg/kg xylazine (both from Bioveta, Czech Republic) in isotonic solution. After decapitation brains were removed and post-fixed for 48h in 4% FA/4°C. Fixed brain hemispheres were dehydrated (in ascending gradients of alcohol), embedded in paraffin, and then cut into 14-µm thick coronal sections using microtome (Leica SM 2000R, Leica Biosystems Nussloch GmbH, Nussloch Germany).

Nissl staining

For analysis of possible structural changes in the brain tissue, cresyl-violet staining, modification by Nissl, was applied on 60-μm and 14-μm coronal sections. The stain 0.5% cresyl violet (Chemika, Girraween, NSW, Australia) in distilled H₂O for 10 min was used. Sections were rinsed in distilled H₂O and dehydrated through ascending concentration of alcohol solutions and cleared with xylene and finally histoclear (Chemika).

Immunofluorescence staining (IF)

For detection of differential expression of specific proteins at P1, immunofluorescent method was applied on free-floating 60μm coronal cryo-sections of the matching anatomical level (Ramachandra and Subramanian, 2011) from hypoxic and control brains. For detection of co-localization of perineuronal nets (PNN) around parvalbumin-expressing interneurons (PV) at P105, immunofluorescent method was utilized on mounted, dewaxed 14µm coronal sections of matching anatomical level (Paxinos and Watson, 2007), from the hypoxic and control brains. Sections were washed for 10 min in PBS, immersed for 1h in blocking solution (3% NDS; 0,01% TRITON/PBS) at room temperature (RT), then incubated with primary antibody (Table 1, except negative control) for 24h/4°C; Yoon et al., 2018), afterwards washed in PBS and incubated with secondary antibody for 2h/RT, and finally sections were rinsed with PBS and mounted on glass slides with Vectashield medium with DAPI (H-1200, Vector Laboratories, USA).

Immunohistochemistry staining (IHC)

Classical immunohistochemical method on free-floating 60μm coronal cryo-sections, of matching anatomical level from the hypoxic and control brains (Ramachandra and Subramanian, 2011) was employed for detection of differential expression of specific proteins at P1. For detection of perineuronal nets around parvalbumin-expressing interneurons at P105, classical histochemical method on mounted and dewaxed 14µm coronal sections of matching anatomical level from the hypoxic and control brains (Paxinos and Watson, 2007), were employed as described previously (Bobić Rasonja et al., 2019). Sections were washed for 3x10 min in PBS and pre-treated in 0.3% hydrogen peroxide in a methanol:water solution in ratio 3:1 for 30 min, washed in PBS for 10 min and immersed into blocking solution (5% BSA; 0.5% TRITON/PBS) at RT for 1h. Sections were incubated with primary antibody (cryo-sections for 24h (Boddaert et al., 2018), paraffin sections for 48h (Ueno et al., 2018a, 2018b) at 4°C, then rinsed in PBS and incubated with appropriate biotinylated secondary antibody for 1h at RT. After washing, sections were incubated in Vectastain ABC reagent (streptavidin-peroxidase complex) at RT for 1h, rinsed in PBS for 10min, and the peroxidase activity was visualized using Ni-3,3-diaminobenzidine (D0426 SIGMAFAST DAB, Sigma, Merck, Germany) for: 5 min (CD68, Parv, Wfa) or 10min (Iba-1). Sections were then rinsed with PBS, dried with Histoclear and mounted with Histomount (National Diagnostics, Atlanta, GA, USA). Negative controls were included in all immunohistochemical experiments by replacing the primary antibody with the blocking solution or pre-immune goat or horse serum, or by omitting the secondary antibody, or replacing it with an inadequate secondary antibody. No immunoreactivity was detected in the control sections.

Microscopy and image processing

The qualitative analysis of the histological sections was performed using an upright Olympus Provis AX70 microscope (Hamburg, Germany) and images were captured with a Nikon DXM1200 digital camera. Immunofluorescent image acquisition was done by confocal Olympus FV3000 microscope (Hamburg, Germany), a 40x objective (UPlanSApo, NA 1.25, FV31S-SW software, and 2048 x 2048 pixels resolution).

Quantification of PNN and parvalbumine-immunoreactive neurons (PV)

The number of PNN and PV neurons were analyzed in coronal sections of the cingulate cortex at the levels bregma -1.56mm to -1.92mm for midcingulate area, and bregma -2.04mm to -2.92mm for retrosplenial area (Paxinos and Watson, 2007). After selection of slides (according to quality of sections, staining intensity and background intensity) 16 animals (9F and 7M) from hypoxic (n=9; 5F and 4M) and control (n=7; 4F and 3M) group were used for quantitative analysis using motorized microscope-computer based system and the Neurolucida software version 10 (MBF–Bioscience, Williston, ND, USA). System was composed of z-axis motorized Olympus BX61 microscope equipped with x-y motorized stage guided by MAC5000 stage controller (Ludl Electronic Products Ltd, Hawthorne, NY, USA). Quantifications for both stainings were made on 3 sections per animal for each area. Then using Neurolucida Explorer software and Markers and Region Analysis plugin results of total area (µm^2^) and total number of PNN or PV were obtained. Data were processed by calculating mean values per animal (N/mm^2^) for each staining. An experimenter was blind to the treatment conditions.

Behavioral testing

Each animal from total of 40 (20F and 20M; hypoxia-treated n=22 /10F and 12M/ and control n=18/ 10F and 8M/) was submitted to a battery of behavioral tests in the following order: open field, hole board, T-maze, social choice, with one day of break between each experiment. The testing was performed in an isolated room, between 1p.m. and 5p.m., under illumination of 30lx and temperature of 21 ± 2°C. Animals were tested in random order by blinded experimenters. Horizontal and vertical locomotor activities were tested in the open field as the total distance covered (TDC, in cm) and the number of rearing (R) during 5 min. Exploratory and thigmotactic behaviors were tested in a hole-board as the total number of holes visited (THV), and the percentage of the inner holes visited (% IN) during 5 min. Learning was tested in a T-maze as a number of correct choices in 10 consecutive trials, during 5 consecutive days. Sociability was tested as the amount of time spent exploring an inanimate object (TO, in second) and a conspecific (TR, in seconds) during 5 minutes. Rats were re-tested according to the same protocol at adult age (P70) as previously described (Blazevic et al., 2012).

Apparatuses

Open field consisted of an 80 cm × 80 cm × 70 cm enclosure made of opaque plexi-glass walls with dark floor. It turned into a hole-board by adding a bottom of white plexi-glass, containing 16 holes (4cm in diameter, 4cm deep), displayed in a 4×4 configuration. The T-maze consisted of a 70cm x 35cm x 10cm central arm and two 55cm x 35cm x 10cm side arms made out of black plexi-glass. The social choice apparatus consisted of a 90cm x 90cm x 50cm black plexiglass enclosure, separated into three equally sized compartments by the two transparent plexiglass barriers with sliding doors.

Procedures

Open field: Animal was placed in the middle of the apparatus and allowed to freely explore during a 5 minutes period. Trials were filmed with a camera placed above the apparatus and videos were later analyzed with EthoVisionXT13 software. Total distance covered reflected horizontal activity, and the number of rearings (upright body with raised front paws, supported or unsupported) reflected vertical activity.

Hole board: Animal was placed in the middle of the apparatus and allowed to freely explore during a 5 minutes period. The number of head dips (both eyes in a hole) in each hole were recorded. Total number of holes reflected exploratory behavior, and the percentage of the visited inner holes reflected thigmotactic (anxiety-like) behavior.

T-maze: Animal was placed at the beginning of the central arm, while the reward (a food pellet) was placed in one of the side arms. Testing started with a zero trial, in which rats passed through a maze once to determine their natural arm selection. During 5 days of learning trials, the reward was placed in the arm opposite to the one the rat had chosen on the zero trial. Each day, rats were given 10 consecutive trials to find the reward. The attempt was recorded as successful if the animal reached the reward within 60 seconds. In order to increase motivation, on the evening preceding the T-maze test, food was removed from the cage and, during the whole testing period, rats had access to food only for one hour daily, after the end of a session.

Social choice: A testing rat was placed into the central compartment, while the side compartments contained an inanimate object and a same-sex conspecific placed in a wire enclosure, respectively. After three minutes of habituation, the side doors opened allowing the rat to freely explore the object or the conspecific (the wire cage allowed olfactory, visual, auditory, and tactile contact with the conspecific, but only the testing animal could initiate the social contact). Time spent exploring the object or the conspecific was recorded during a 5 minutes period. Testing apparatuses were washed with detergent, rinsed with water, and dried after each animal, or after each trial.

**Supplementary Figure 1.** A representative image of total protein signal on the membrane after transfer using No-Stain protein Labeling reagent (#A44449).


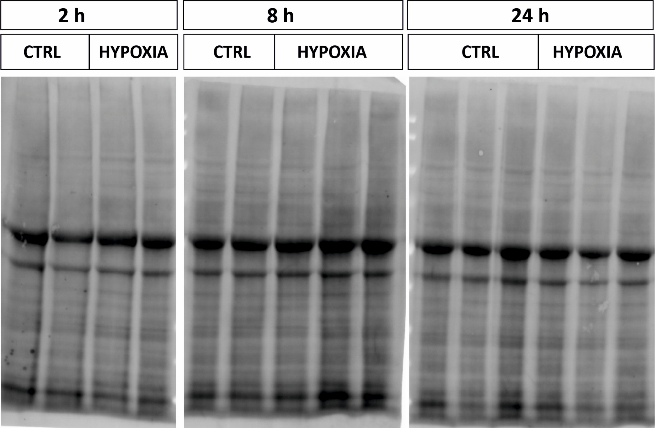


**Supplementary References**

Blazevic, S., Colic, L., Culig, L., and Hranilovic, D. (2012). Anxiety-like behavior and cognitive flexibility in adult rats perinatally exposed to increased serotonin concentrations. *Behav. Brain Res.* 230, 175–181. doi:10.1016/j.bbr.2012.02.001.

Bobić Rasonja, M., Orešković, D., Knezović, V., Pogledić, I., Pupačić, D., Vukšić, M., et al. (2019). Histological and MRI Study of the Development of the Human Indusium Griseum. *Cereb. Cortex* 29, 4709–4724. doi:10.1093/cercor/bhz004.

Boddaert, J., Bielen, K., ’s Jongers, B., Manocha, E., Yperzeele, L., Cras, P., et al. (2018). CD8 signaling in microglia/macrophage M1 polarization in a rat model of cerebral ischemia. *PLoS One* 13, e0186937. doi:10.1371/journal.pone.0186937.

Chen, W., Jadhav, V., Tang, J., and Zhang, J. H. (2008). HIF-1α inhibition ameliorates neonatal brain injury in a rat pup hypoxic–ischemic model. *Neurobiol. Dis.* 31, 433–441. doi:10.1016/j.nbd.2008.05.020.

Deng, C., Li, J., Li, L., Sun, F., and Xie, J. (2019). Effects of hypoxia ischemia on caspase-3 expression and neuronal apoptosis in the brain of neonatal mice. *Exp. Ther. Med.* 17, 4517–4521. doi:10.3892/etm.2019.7487.

Gage, G. J., Kipke, D. R., and Shain, W. (2012). Whole Animal Perfusion Fixation for Rodents. *J. Vis. Exp.*, 1–12. doi:10.3791/3564.

Li, Y., Park, J.-S., Deng, J.-H., and Bai, Y. (2006). Cytochrome c oxidase subunit IV is essential for assembly and respiratory function of the enzyme complex. *J. Bioenerg. Biomembr.* 38, 283–291. doi:10.1007/s10863-006-9052-z.

Paxinos, G., and Watson, C. (2007). *The Rat Brain in Stereotaxic Coordinates 6th Edition*. Amsterdam; Boston: Elsevier Academic Press.

Phifer, C. B., and Terry, L. M. (1986). Use of hypothermia for general anesthesia in preweanling rodents. *Physiol. Behav.* 38, 887–890. doi:10.1016/0031-9384(86)90058-2.

Ramachandra, R., and Subramanian, T. (2011). “Coronal Plates,” in *Atlas of the Neonatal Rat Brain* (CRC Press), 3–32. doi:10.1201/b10500-3.

Rivero-Gutiérrez, B., Anzola, A., Martínez-Augustin, O., and de Medina, F. S. (2014). Stain-free detection as loading control alternative to Ponceau and housekeeping protein immunodetection in Western blotting. *Anal. Biochem.* 467, 1–3. doi:10.1016/j.ab.2014.08.027.

Ueno, H., Suemitsu, S., Murakami, S., Kitamura, N., Wani, K., Matsumoto, Y., et al. (2018a). Hyaluronic acid is present on specific perineuronal nets in the mouse cerebral cortex. *Brain Res.* 1698, 139–150. doi:10.1016/j.brainres.2018.08.011.

Ueno, H., Takao, K., Suemitsu, S., Murakami, S., Kitamura, N., Wani, K., et al. (2018b). Age-dependent and region-specific alteration of parvalbumin neurons and perineuronal nets in the mouse cerebral cortex. *Neurochem. Int.* 112, 59–70. doi:10.1016/j.neuint.2017.11.001.

Yoon, J. S., Jo, D., Lee, H.-S., Yoo, S.-W., Lee, T.-Y., Hwang, W. S., et al. (2018). Spatiotemporal Protein Atlas of Cell Death-Related Molecules in the Rat MCAO Stroke Model. *Exp. Neurobiol.* 27, 287–298. doi:10.5607/en.2018.27.4.287.
